# A Cone-Beam Computed Tomographic Analysis of Mesiobuccal Root Canals of Maxillary First Molars

**DOI:** 10.7759/cureus.46110

**Published:** 2023-09-28

**Authors:** Ashwaq F Asiri

**Affiliations:** 1 Department of Restorative Dental Sciences, College of Dentistry, Majmaah University, Majmaah, SAU

**Keywords:** cone beam computed tomography, isthmus, vertucci’s classification, mesiobuccal root canal, maxillary first molar

## Abstract

Introduction: The maxillary first molar is crucial for proper bite formation and jaw positioning in adulthood. The prevalence of dental caries in the study's sample population suggests it is a common candidate for endodontic therapy. Multiple studies have shown that the canal and root morphology of the maxillary first molar are abnormal.

Materials and methods:The distobuccal and palatal roots of 286 maxillary first molars were removed at the furcation. The mesiobuccal roots were then imaged using cone-beam computed tomography (CBCT) on all specimens. The specimens were analyzed in comparison with one another. The following factors were studied. The number and arrangement of canals, as described by Vertucci; the presence or absence of an isthmus, as described by Kim; the canal curvature angle, as measured by the Schneider method; characteristics such as calcified segments, lateral canals, and an apical delta. For the flow analysis, CBCT was utilized to examine the root channel architecture of 286 mesiobuccal (MB) permanent maxillary first molar.

Results: Type I canal arrangement was found in 51.6% of teeth, Type II in 33.3%, Type III in 4.6%, Type IV in 4.2%, Type V in 2.5%, and Type VII in 1.5%. Type I, II, III, and V isthmus were each present in 26%, 6.7%, 9.5%, and 10.2% of the samples, respectively. Teeth with MB1 canal angulations of 0-20 degrees, 21-40 degrees, and more than 40 degrees were found in 56, 188, and 41 teeth, respectively. From 0 to 20 degrees, 21 to 40 degrees, and more than 40 degrees, 15 teeth, 88 teeth, and 25 teeth, respectively, had angulations in their MB2 canals. Only 9.8% of the samples had lateral canals, while 16.1% had both accessory canals and apical deltas. There were 19 cases with calcified segments in the coronal third of the MB1 canal and 13 cases in the middle third. There were no calcifications at the distal end of the MB1 canal. Thirteen of the specimens showed calcification only in the most caudal third of the MB2 canal, whereas the middle and distal thirds were uncalcified.

Conclusion: Utilizing a noninvasive approach, a CBCT scan has the capacity to provide valuable insights into the root canal configuration.

## Introduction

A successful root canal treatment outcome is achieved by incorporating strict isolation techniques, thorough chemomechanical preparation, and rigorous three-dimensional obturation. The maxillary first molar usually emerges before any other permanent teeth. It is also one of the most susceptible to dental caries. It's an essential part of understanding occlusion. Loss of the first permanent molar in the upper jaw too soon might compromise the stability of the arch and cause malocclusion [1,2].

Two canals in the mesiobuccal (MB) root were found to be 56.8% common and one canal to be 43.1% common in a review of the literature conducted by Cleghorn et al. [3]. This intricate canal anatomy has been investigated using a wide range of methods. Clinical investigations in real life have included looking back at patient charts, analyzing x-rays, and doing in-person exams of teeth during endodontic procedures with and without magnification. The employment of India ink, Chinese ink, hematoxylin dye, and other colors on extracted teeth has been the subject of various clearance experiments [4]. These teeth have also been used for radiography, scanning electron microscopy, tooth sectioning, and endodontic access and examination in in vitro laboratories.

Second mesiobuccal canals (MB2) in maxillary molars have been documented at a frequency ranging from 10% to 95%. Recent research has also shown that certain maxillary first molars have a third mesiobuccal canal, with an incidence of roughly 7% [4]. Peters et al. cited Hitoshi Tachibana's 1990 research, in which he used computed tomography (CT) scans to examine the anatomical arrangements of teeth [5]. Recently, a thorough three-dimensional investigation of the root canal system was carried out by combining mathematical modeling with micro-computed tomography (microCT) [4]. In this study, cone-beam computed tomography (CBCT) was employed to extraorally investigate the mesiobuccal canals of maxillary first molars.

## Materials and methods

**Figure 1:**
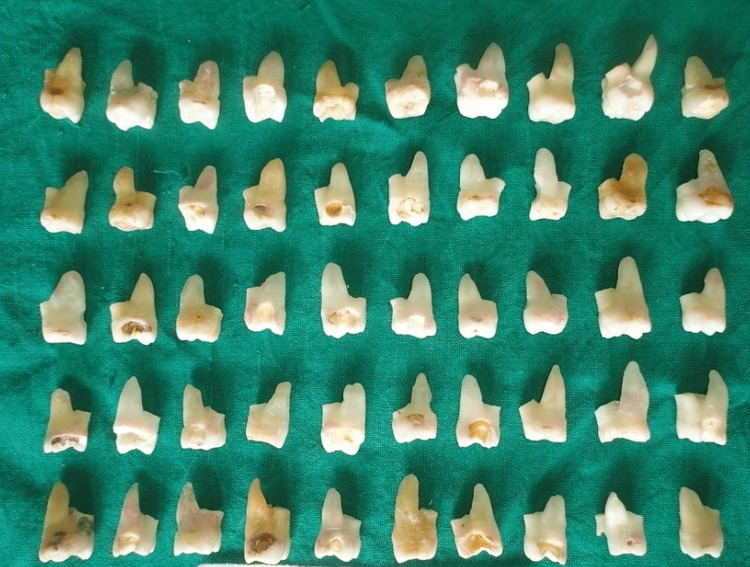
Specimens used

Patients over the age of 18 who had visited the Department of Oral Surgery for extractions owing to severe caries or periodontal disease provided a total of 286 maxillary first molars with intact mesiobuccal roots for analysis. The research did not include teeth that had fillings or crowns (Figure 1).

Using an ultrasonic scaler, the dentist carefully removed calculus and soft tissue attachments from the teeth's surface before soaking them in a solution of sodium hypochlorite (5.25%). To preserve the tooth specimen, a carborundum disc was used to sever the distobuccal and palatal roots at their furcation, and 0.2% sodium azide was added to the distilled water. We used absorbent paper to dry the samples before storing them and scanning them. They were transported to the Insight CBCT Scanning Centre, where a CBCT scan was done on them after they had been laid out on a wax sheet. No more than six specimens were anchored to any one wax sheet. This methodical approach was undertaken to minimize overlapping, distortion, or any potential interference during the scanning process.

Cone-beam computed tomography

**Figure 2:**
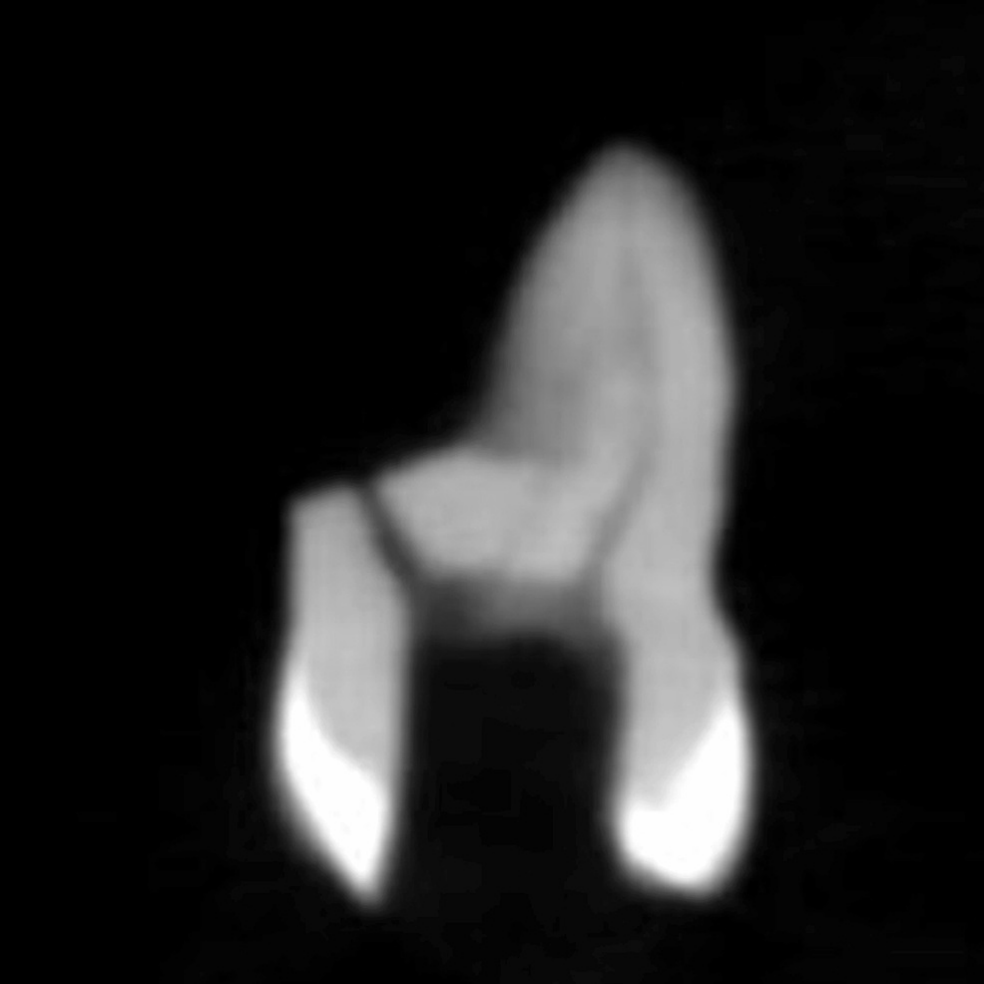
Cone-beam computed tomography (CBCT) scan of the tooth in the study for lateral canal evaluation

**Figure 3:**
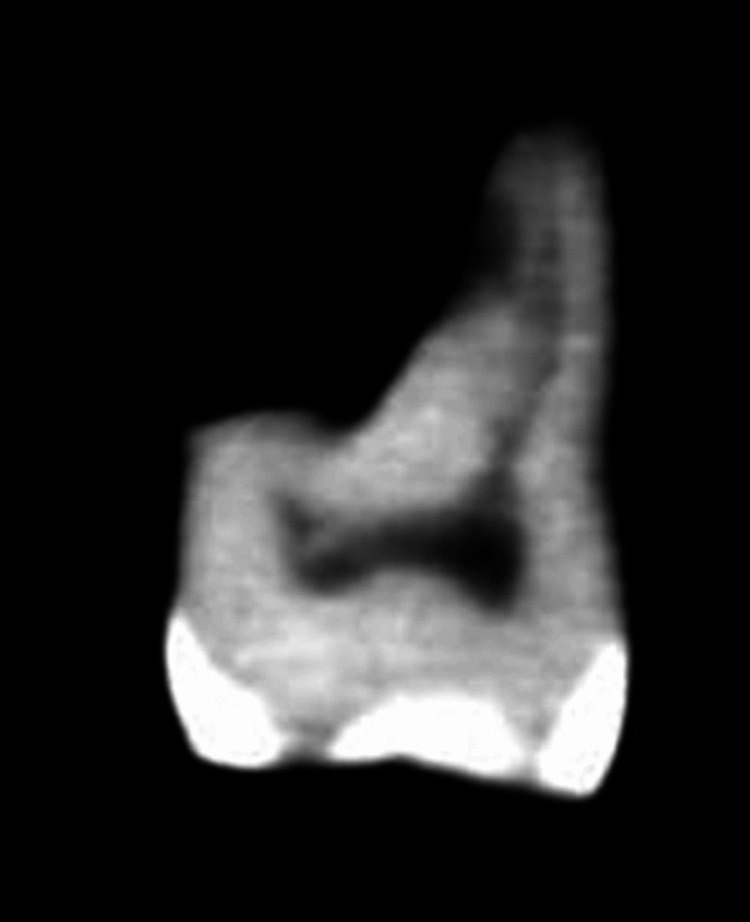
Cone-beam computed tomography (CBCT) scan of the tooth in the study for lateral canal evaluation

**Figure 4:**
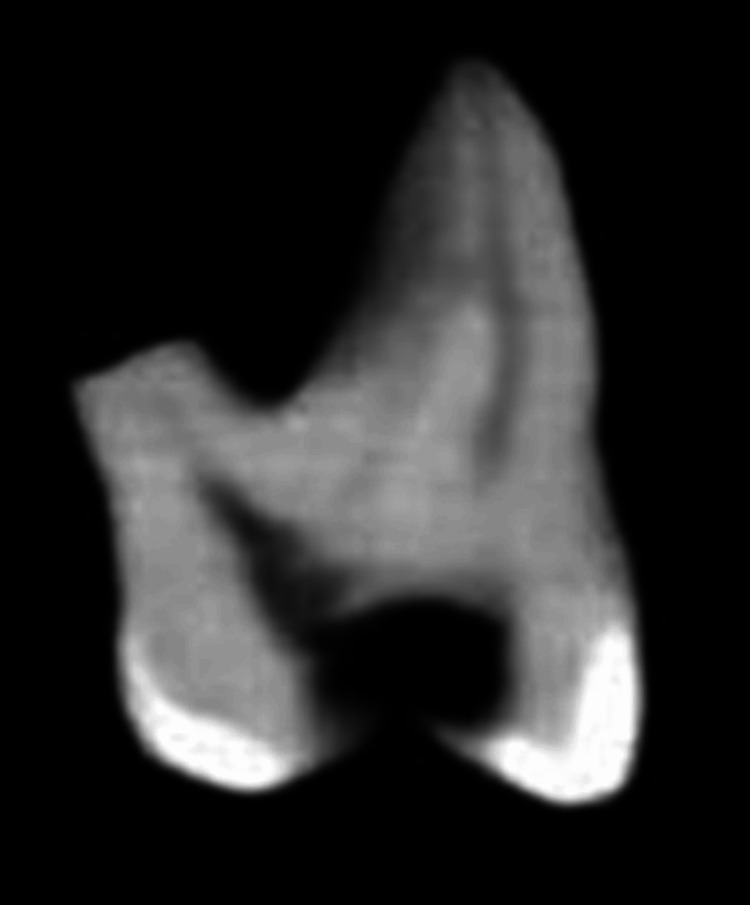
Cone-beam computed tomography (CBCT) scan of the tooth in the study

An external imaging scanner is required for CBCT. By synchronizing the rotation of the sensor with the radiation source, CBCT may gather a three-dimensional volume of data with a single scan of the patient's head. CBCT scans from the i-CAT Cone Beam 3-D Dental Imaging System take about 30 seconds on average. There may be as many as 580 unique “small openings” or “projection pictures” captured in the output, despite the fact that the actual open duration is just 2-5 seconds due to the X-beam pillar beating. CBCT was used to scan the teeth, and slices were taken to create pictures of each tooth. A uniform 125 m/slice was used to scan all of the teeth. Axial (offering a comprehensive view of the tooth's cross-section at the respective levels), coronal (offering insights into its internal structure at different vertical positions), and sagittal sections (this view provides a lateral perspective of the tooth's structure) were taken at the furcation, midroot, and apical levels, and the resulting pictures were captured (Figures 2, 3, 4).

Classification of canal configuration

**Figure 5:**
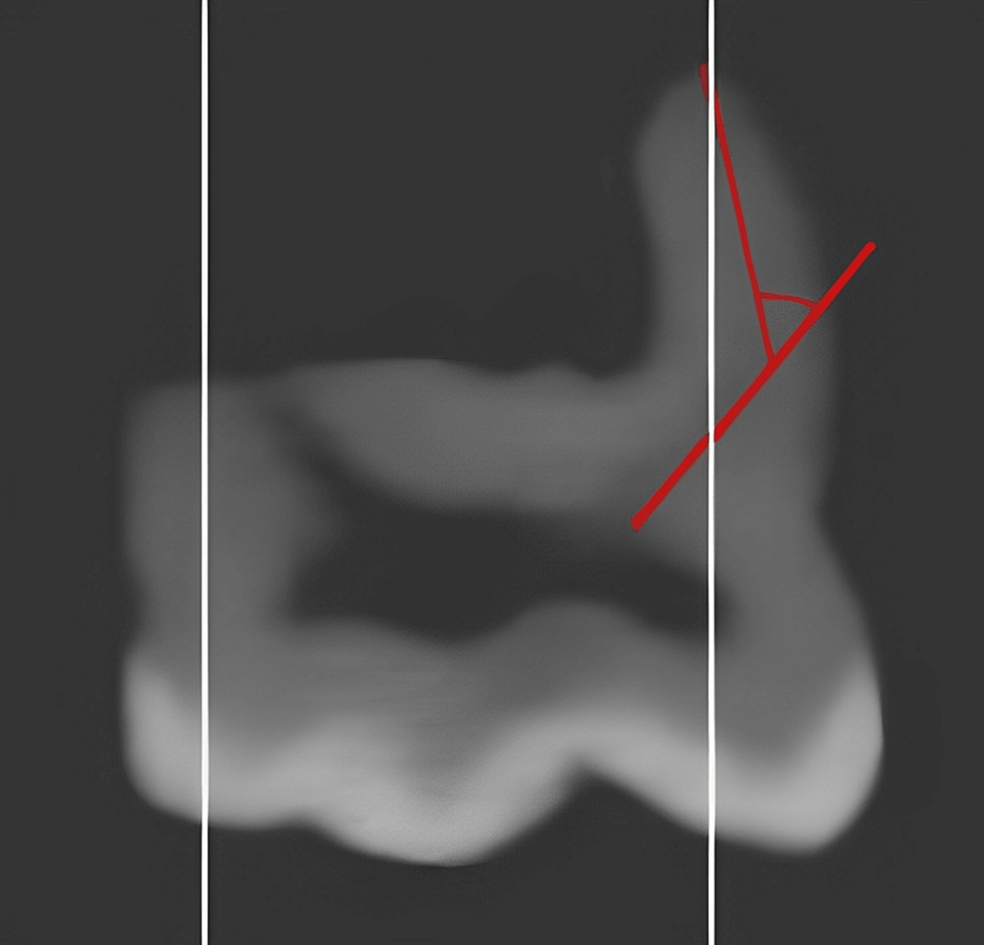
Canal's curvature calculated using Schneider's classification

Each maxillary first molar's MB root canal system was meticulously analyzed by analyzing its recorded photos. By carefully inspecting for branching only at the cervical or middle third, the canal system was planned out, and each MB canal network was assigned a classification according to Vertucci's approach. Auxiliary canals are those that branch out from the main canal fewer than three millimeters from the point of the tooth's apex. If there was an isthmus in a tooth, it was identified and categorized according to Kim's system. To ensure that the whole length of each canal was captured, appropriate picture slices were chosen. A central axis was created by manually plotting a major and minor axis on each slice and connecting their intersections. Each canal's curvature was calculated using Schneider's classification (Figure 5).

Each tooth was meticulously sectioned in order to examine its apex and lateral canals. We looked at calcifications in the first, second, and third thirds of each canal's length. Differential diagnoses, if any, were recorded. 

## Results

The specimens in this research were evaluated using the Vertucci categorization system, and the following characteristics (I-V) were analyzed and tallied. The research conducted a comprehensive investigation into the root canal anatomy and characteristics of the MB root of the maxillary first molar. To classify the canal patterns, the researchers employed the Vertucci categorization system and found that Type I canal pattern was the most prevalent (51.6%), followed by Type II (33.3%), while the remaining types were less common (Table 1).

**Table 1:** Root canal anatomy of the mesiobuccal root of the maxillary first molar

Canal pattern (Vertucci’s classification)	Number of teeth	Percent
I	147	51.6
II	95	33.3
III	11	3.9
IV	13	4.6
V	12	4.2
VI	7	2.5

In assessing the type of isthmus, Kim's classification was utilized, revealing the prevalence of Type I isthmus (26.0%) as the most frequent, with the other types showing varying occurrences (Table 2).

**Table 2:** Characteristic isthmus of the mesiobuccal root of the first maxillary molar

Type of isthmus (Kim’s classification)	Number of teeth	Percent
I	74	26.0
II	19	6.7
III	27	9.5
IV	5	1.8
V	29	10.2

Schneider's technique was employed to measure the angle of curvature in the MB region, demonstrating a majority of teeth falling within the 210-400-degree range. Additionally, the presence of lateral canals and accessory canals/apical delta in the MB root was analyzed, and it was found that a notable percentage exhibited these features (Table 3).

**Table 3:** Root canal angulation in the mesiobuccal region of the first maxillary molar MB: Mesiobuccal canal

Angle of curvature (Schneider’s technique)	MB1	MB2
0^0 ^– 20^0^	56 (19.6%)	15 (5.3%)
21^0 ^– 40^0^	188 (66%)	88 (30.9%)
Greater than 40^0^	41 (14.4%)	25 (8.8%)

Furthermore, the study investigated the presence of calcified segments in the MB1 and MB2 roots, revealing the distribution across the coronal and middle thirds. Overall, these findings offer valuable insights into the intricate root canal morphology of the maxillary first molar, providing crucial information that can aid dental professionals in their endodontic treatments and enhance their understanding of this complex dental structure (Table 4, 5).

**Table 4:** The first maxillary molar has a mesiobuccal root with apical delta, lateral canals, and accessory canals

Variables	Number of teeth	Percent
Lateral canals	28	9.8
Accessory canals and apical delta	46	16.1

**Table 5:** Roots of the upper right first molar, at the mesiobuccal aspect, have developed calcified segments MB: Mesiobuccal canal

Calcified segment	Coronal third	Middle third	Apical third
MB1	19	13	0
MB2	13	4	0

## Discussion

The anatomical intricacy of root canals has much to do with the persistence of bacteria. Untreated canal ramifications and isthmus, as well as improper chemo-mechanical preparation and root canal filling, all help keep the infection going, which is what happens in the root canal. According to the literature, the incidence of C-shaped canals in maxillary first molars is 0.12% [6], making this aberration very uncommon. The root of the distobuccal bone might merge with the palatal bone, or the distobuccal bone can fuse with the mesiobuccal bone. The first maxillary molar's mesiobuccal root could have one, two, or even three canals. Due to their buccolingual breadth and concavities on both the mesial and distal sides, mesiobuccal roots have two canals, whereas distobuccal and palatal roots have only one canal. Upper first molar pulp chambers have a rhomboid form with rounded corners.

When seen from the side, the pulp chamber has a steep buccal and mesial angle to the distobuccal opening. houses the primary orifice of the mesiobuccal canal (MB1). When the opening of the palatal canal is located palatally. If there is more than one mesiobuccal canal, the second one's orifice (MB2) will be palatal and mesial to the first (MB1) [7]. Based on my clinical observations, the MB2 canal of the maxillary first molar seems to be the most elusive and challenging to access. The canal system seen in the molars of the molar group (MB) of Caucasian teeth has been the primary focus of most studies. which may account for some of the discrepancies seen in the literature. The literature reports on only a few number of investigations on the canal architecture of maxillary teeth in Asian ethnicities. The Indian population is sometimes described as belonging to a racial mixture known as the Dravidians, which includes elements of the Caucasian, Mongoloid, and Negroid races. Neelakantan et al. studied an Indian population to learn more about the root and canal anatomy of the first and second maxillary molars [8]. The MB root was discovered to have partially clogged canals as a result of aging and calcification. Among males, only 3% had a single Vertucci type I canal in the mesiobuccal root, compared to 10% among females [9].

When compared to conventional radiographic technique (periapical radiographs, bitewing radiographs), CBCT has several advantages. Periapical pathologies could be diagnosed earlier using CBCT and providing insight into the true size, extent, kind, and location of periapical and resorptive lesions. The shape of the alveolar bone that surrounds teeth may be inferred from root fractures and the structure of root canals. CBCT investigations may be hindered by scatter and beam hardening brought on by surrounding high-density materials like enamel, metal posts, and repair. As a result, those who had recently had dental work like fillings or crowns were excluded. Although Sert and Bayirli [10] proposed some tweaks, the current study relied on the Vertucci classification, which is still the gold standard. Fifty-one percent (147 teeth) of the 286 specimens studied fit the type I pattern. 33.3 percent (95 teeth) had a type II pattern. There was a type III canal pattern in 3.9% (11 teeth). Type IV canal pattern was seen in 4.6% (13 teeth). Type V canal patterns were found in 4.2% (12 teeth) and 2.5% (seven teeth), whereas type VI canal patterns were found in 2.5%.

In the mesiobuccal root of the maxillary first molar, type I canal configuration accounts for 39.3% and type IV for 23.7%, as previously studied by Pineda and Kuttler [11]. Pecora et al. [12] observed that 75% of canals were configured as type I, whereas 17.5% were configured as type II. Weine et al. revealed 42% type I canals and 30.45% type IV canals [13], whereas Neelakantan et al. discovered 51.8% type I configurations and 38.6% type IV topologies [8]. The canal patterns in the current study's specimens are comparable to those found in Pecora et al. [12] (mostly types I and II).

One research suggested using methylene blue dye to help see the border of the resected root surface and spot an isthmus [7]. The effectiveness of surgical treatments relies on isthmi being identified and treated. There are five distinct forms of isthmi that may appear on a root's beveled surface, as described by Kim et al. [14]. Between 4 and 6 millimeters (mm) away from the apical foramen is where an isthmus is seen in the majority of instances (89%). In this sample, 26.4% of teeth had an isthmus of type I, 6.7% had an isthmus of type II, 9.5% had an isthmus of type III, 1.8% had an isthmus of type IV, and 10.2% had an isthmus of type V. In this analysis, most isthmi were located in the cervical and middle thirds of roots, around 6-7 mm from the root tip. This contradicts prior research that found most canal isthmi to occur around the root's tip. Based on the findings of this research, it seems more likely that isthmi is an ethnic feature shared by Indians. The localization, chemomechanical preparation, and obturation of the MB2 canal may be a more effective orthograde root canal therapy for controlling these isthmi at a more apical level in the mesiobuccal root. Several methods exist for creating canals that are both larger and more uniform in size. The problem gets considerably more difficult when the canal curvature is more than 30 degrees, and solutions that work well in simpler scenarios may or may not work in more complex instances [7]. Schneider explains that a radiograph is used to make this kind of diagnosis [9].

Among the samples in this study, 56 had an MB1 angle between 0 and 20 degrees, 188 had an MB1 angle between 21 and 40 degrees, and 41 had an MB1 angle greater than 40 degrees. Fifteen specimens had an MB2 canal angle of 0°-20°, 88 had an MB2 canal angle of 21°-40°, and 25 had an MB2 canal angle of 40° or more. Importantly, after each file is used in the dependent canal, to guarantee apical patency, the file must travel the complete length of the master canal. A tooth with a Type V canal system has a single canal that branches off into two canals at the apex of the tooth, making preparation more complicated.

The root's length is not always related to the presence of lateral or auxiliary canals. Approximately one-third of all tooth roots, near the root tip, contain lateral or accessory canals. Nearly 9% of the teeth studied by De Deus had lateral canals in the central third of the root, and fewer than 2% of lateral canals were linked to periodontal pockets. The presence of radiolucency away from the root's apex indicates the presence of a large lateral canal or a laterally located apical foramen. In nonsurgical circumstances, lateral canals are often detected by the extrusion of sealer on the post operation radiograph [14]. Nine percent of the specimens in this research (28 teeth) had lateral canals, whereas 16% (46 teeth) had accessory canals and an apical delta.

The pulp and the periodontium are connected by tiny canals called accessory canals. These canals may run horizontally, vertically, or laterally. In terms of pathology, they are important because they allow irritants to move freely between the pulp and the periodontium [7]. This study found that the most calcified areas of MB2 canals were located in their dorsal (upper) regions. The dorsal one-third of the MB1 canal had the largest concentration of calcified segments, followed by the middle one-third (13) and the dorsal one-third (13) of the MB2 canal (4). However, calcified segments in the MB2 canal's coronal third were found in 13 of the cases. Periodontal arteries get trapped in the calcifying epithelial root sheath of Hertwig's tooth [7]. After coronal dentin was removed (via ultrasonic troughing), Yoshioka et al. found numerous additional MB2 canals. It was also hypothesized [15] that calcifications prevented the detection of several MB2 canals. The mesiobuccal root canals of the maxillary first molar have been the subject of a number of prior research and examinations [16-18].

Additional research is needed to verify the findings of the current study. However, demographic factors such as age, sex, race, etc. were not accounted for in this study. As more studies are conducted, the mesiobuccal root of the maxillary first molars is where we might anticipate learning more about the canal layout.

## Conclusions

This study examined the MB root of maxillary first molars and found that the Vertucci classification system identified Type I and Type II canal arrangements as the most common, while Type III, Type IV, Type V, and Type VII were also observed, albeit with lower frequencies. The investigation of isthmus types using Kim's classification revealed variations, with Type I being the most prevalent. Additionally, the study analyzed the angle of curvature in MB1 and MB2 canals, the presence of lateral and accessory canals/apical deltas, and calcified segments in the roots. Overall, the research highlights the potential of noninvasive cone-beam CT scans with minimal radiation for better understanding of root canal configurations in maxillary first molars. These findings contribute to our knowledge of endodontic anatomy and can assist dental practitioners in optimizing treatment approaches for these complex dental structures.
